# Mathematical Modeling and Experimental Substantiation of the Gas Release Process in the Production of Non-Autoclaved Aerated Concrete

**DOI:** 10.3390/ma15072642

**Published:** 2022-04-03

**Authors:** Evgenii M. Shcherban’, Sergey A. Stel’makh, Alexey Beskopylny, Levon R. Mailyan, Besarion Meskhi, Anatoly Shuyskiy, Nikita Beskopylny, Natal’ya Dotsenko

**Affiliations:** 1 Department of Engineering Geology, Bases and Foundations, Don State Technical University, 344003 Rostov-on-Don, Russia; au-geen@mail.ru (E.M.S.); sergej.stelmax@mail.ru (S.A.S.); 2 Department of Transport Systems, Don State Technical University, 344003 Rostov-on-Don, Russia; 3 Department of Roads, Don State Technical University, 344003 Rostov-on-Don, Russia; lrm@aaanet.ru; 4 Department of Life Safety and Environmental Protection, Don State Technical University, 344003 Rostov-on-Don, Russia; reception@donstu.ru; 5 Department of Technological Engineering and Expertise in the Construction Industry, Don State Technical University, 344003 Rostov-on-Don, Russia; a2293613@mail.ru (A.S.); natalya_1998_dotsenko@mail.ru (N.D.); 6 Department Hardware and Software Engineering, Don State Technical University, 344003 Rostov-on-Don, Russia; beskna@yandex.ru

**Keywords:** aerated concrete, gassing, swelling, blowing agent, average density, thermal conductivity

## Abstract

The widespread use of aerated concrete in construction has led to the emergence of many types and compositions. However, additional research should fill theoretical gaps in the phenomenon of gas release during the formation of the structure of aerated concrete. Based on theoretical analysis and experimental studies, the article proposes a mathematical model of the swelling process based on the physicochemical laws of convection and molecular diffusion of hydrogen from a mixture and the conditions of swelling, precipitation, and stabilization of the mixture. An improved method for the manufacture of aerated concrete is proposed, which consists of introducing cement pre-hydrated for 20–30 min into the composition of the aerated concrete mixture and providing improved gas-holding capacity and increased swelling of the mixture, reducing the average density of aerated concrete up to 29% and improving heat-shielding properties up to 31%. At the same time, the small dynamics of a decrease in the strength properties of aerated concrete were observed, which is confirmed by an increased structural quality factor (CSQ) of up to 13%. As a result, aerated concrete has been obtained that meets the requirements of environmental friendliness and has improved mechanical and physical characteristics. Economic efficiency is to reduce the cost of production of aerated concrete and construction in general by about 15%.

## 1. Introduction

### 1.1. Relevance and Article Tasks

The study’s relevance is due to the current scientific lack of research on the fundamental nature and substantiation of mathematical and physical models to form promising cellular concrete. This material fully meets the world’s requirements for energy efficiency and energy saving. It is a suitable material for improving its porous structure with the help of formulation and technological factors to obtain the most efficient building structures with a minimum cross-section and minimum thermal conductivity. In terms of cost characteristics, non-autoclaved cellular concretes significantly outperform autoclaved competitors due to the significantly reduced cost of production and manufacture of such concretes and less environmental impact. From the point of view of technological and recipe factors, there are quite a lot of studies of non-autoclaved and autoclaved aerated concrete [1,2,3,4,5,6,7,8,9,10,11,12,13,14,15,16,17,18,19,20,21,22,23,24,25,26,27,28,29,30,31,32,33,34,35]. However, from the point of view of fundamental science, some gaps should be filled with additional research in the phenomenon of outgassing during the formation of the structure of non-autoclaved aerated concrete.

Studies [2,3,4] are devoted to the possibility of obtaining aerated concrete products using iron and graphite tailings. The influence of the content and size of iron tailings on the mechanical properties of aerated concrete products has been studied, the products of hydrothermal synthesis of aerated concrete have been considered, and new types of autoclaved aerated concrete have been developed. The production of autoclaved aerated concrete (AAC) using graphite tailings such as silicon is described in [4]. In addition to cement dosage, water to solids ratio, and foaming agent content, the calcium–silicon ratio also plays an essential role in producing graphite waste autoclaved aerated concrete (GT-AAC).

It is known that the recycling of aerated concrete is widely used as raw material in the production of aerated concrete products and structures [5,6,7,8,9,10]. Thus, it was found in [5] that when replacing sand with recycled aerated concrete, an increase in compressive strength is 16% higher than that of conventional aerated concrete and 29–156% higher than the values obtained using industrial waste.

Studies of the influence of various fibers and microsilica on lightweight concrete’s mechanical properties (compressive and bending strength) are also of great importance [11,12,13,14,15,16]. For example, in [11], the change in the value of thermal conductivity, compressive strength, and bending of autoclaved aerated concrete was studied when polypropylene, carbon, basalt, and glass fibers were added to autoclaved aerated concrete. It was found that the thermal conductivity of AAC with the addition of fibers varies linearly with the thermal conductivity of the fiber and that AAC reinforced with basalt fiber gives the highest thermal conductivity. In addition, the influence of the type and size of the fiber in aerated concrete production, on the values of compressive strength, bending, and thermal conductivity was studied [14]. Aerated concrete samples were made with polypropylene, carbon, basalt, and glass fibers. The study [14] found that adding various reinforcing fibers to aerated concrete resulted in increased flexural and compressive strength, and carbon fiber reinforced aerated concrete gave better flexural and compressive strength than other fiber types.

Complex additives are also used in aerated concrete technology. In [15], the authors studied the effect of a complex additive consisting of basalt fibers and SiO_2_ microdust on the strength properties of samples of autoclaved aerated concrete. Based on the results of the studies, it was found that the complex additive affects the process of hydration of cellular concrete. Namely, it leads to calcium hydro silicates of the tobermorite group (C–S–H). The compressive strength of aerated concrete samples containing a complex additive increased by 52%, and the tensile strength in bending by 62% compared with the strength characteristics of samples of the control composition without the additive.

In other studies [17,18,19,20,21,22,23], the authors investigated the thermal conductivity of cellular concrete and various factors affecting it. For example, in [18], the authors developed a model for predicting the thermal conductivity of non-autoclaved cellular concrete using the linearization approach. This model has shown good reliability for predicting the thermal conductivity of cellular non-autoclaved concrete at 28 days of age. It has also been found that the main factors affecting thermal conductivity are the water–cement ratio, curing age, and temperature.

Investigation of dependences and modeling parameters of the behavior of AAC products in terms of wind load [24], material moisture [25,26], strength obtained using non-destructive testing methods [26], replacing aluminum powder on natural zeolite [27], as well as studies on porosity parameters [28,29,30], made a significant contribution to the development of this study and helped to formulate its purpose and scientific novelty.

Several works [31,32,33,34,35] are devoted to the macrostructure formation stage, the analysis of which shows that the determining influence on the formation of the porous structure of aerated concrete is exerted by the kinetics of gas release by the gasifier and the change in the rheological properties of the interpore material. Furthermore, studies of the swelling process to improve the quality of the macrostructure and improve the physical and mechanical properties of aerated concrete are currently aimed at an in-depth study and determination of the optimal combination of these kinetic processes.

### 1.2. Plan, Main Aim, and Hypothesis of the Study

Diffusion phenomena that occur during swelling, as a result of which part of the hydrogen is removed from the cellular concrete mixture, are the consequence of defect formation and lead to an increase in the average density of cellular concrete and a decrease in the quality of products. Therefore, the creation of conditions under which the diffusion phenomena of the intumescent mixture are manifested to a lesser extent makes it possible to achieve relatively better characteristics of the material structure.

**Figure 1:**
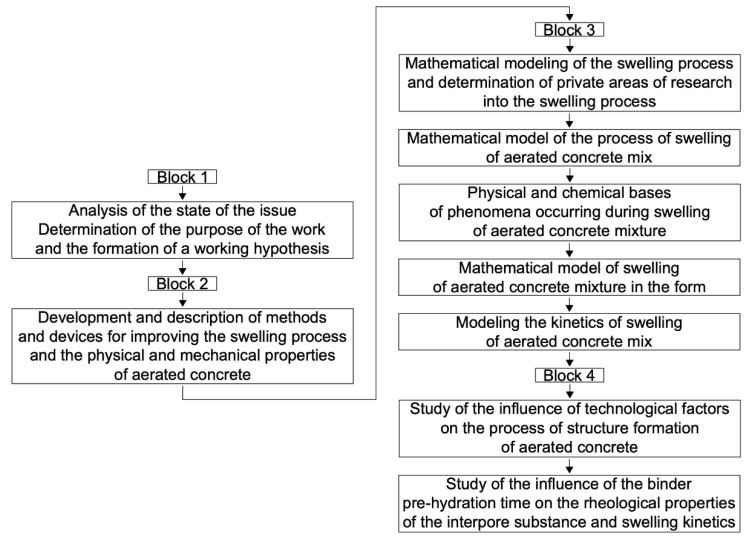
Structural and logical block diagram of the study plan.

The research plan is presented in a structural–logical block diagram (Figure 1).

This work aims to optimize the technological process for the manufacture of aerated concrete products using injection technology at the molding stage, ensuring a decrease in the average density and an increase in the uniformity of material properties. This goal is achieved by deepening the theoretical understanding of the processes of formation of a cellular structure and by optimizing the kinetics of processes occurring during swelling and improving on this basis the technology for manufacturing aerated concrete products, improving the means of controlling the properties of materials.

The working hypothesis of the work is formulated as follows: the intumescent aerated concrete mixture meets the optimal conditions for the manufacture of aerated concrete with desired properties, which ensures the coincidence in time of the start of coagulation of new cement formations and the start of intensive gas release by the aluminum blowing agent.

Based on the mathematical model, it is necessary to calculate an environmentally friendly, economic non-autoclaved aerated concrete that meets the requirements of the UN global ESG agenda for environmental protection, has an improved structure and improved energy-efficient and energy-saving properties, and better mechanical characteristics.

## 2. Materials and Methods

### 2.1. Materials, Test Equipment, and Measuring Instruments

This section lists the characteristics of the raw materials used in the experimental studies. The study of the processes of formation of a cellular structure, the determination of the optimal compositions of cellular concrete, and the physical and mechanical properties of the obtained materials were carried out on cement–sand mixtures.

When carrying out the experiments, Portland cement of the CEM I 42.5N brand produced by JSC Novoroscement (Novorossiysk, Russia) was used. Table 1 presents Portland cement’s physical and mechanical characteristics and its chemical composition.

Quartz sand produced by Arkhipovsky Quarry OJSC (village Arkhipovskoye, Belorechensky District, Krasnodar Territory, Russia) was used as a fine aggregate, the physical characteristics of which are presented in Table 2.

Aluminum powder PAP-1 produced by OOO SKIF (St. Petersburg, Russia) was used as a blowing agent. The physical characteristics and chemical composition of aluminum powder are shown in Table 3.

The compressive strength was determined on 100 mm × 100 mm × 100 mm cube samples following the requirements of GOST 10,180 “Concretes. Methods for strength determination using reference specimens” [36]. The average density of aerated concrete was also determined on cube samples measuring 100 mm × 100 mm × 100 mm by the requirements of GOST 12,730.1 “Concretes. Methods of determination of density” [37].

**Figure 2:**
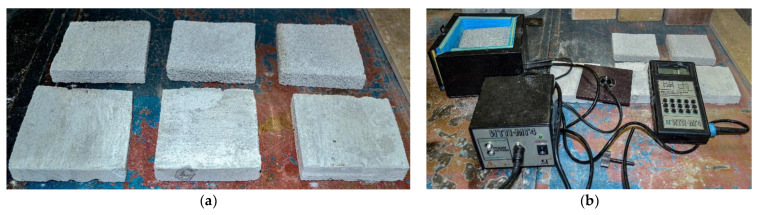
Specimens (**a**) and device (**b**) for testing aerated concrete for thermal conductivity.

The thermal conductivity of aerated concrete was determined under the requirements of GOST 7076 [38] on samples of dimensions 100 mm × 100 mm × 20 mm (Figure 2a).

The study used:

- Technological equipment—installation for the production of foam concrete and aerated concrete GBS-250 manufactured by METEM (Perm, Russia); ball planetary mill “Activator-4M” (LLC “Plant of Chemical Engineering”, Novosibirsk, Russia);
- Testing equipment—hydraulic press MIP-25 (LLC NPP INTERPRIBOR, Chelyabinsk, Russia);
- Measuring instruments—metal measuring ruler 500 mm (JSC “Stavropol Tool Plant”, Stavropol, Russia); laboratory scales HT-5000 (NPP Gosmetr, St. Petersburg, Russia); caliper ShTs-I-250-0.05 (LLC NPP Chelyabinsk Tool Plant, Chelyabinsk, Russia); thermal conductivity meter ITP-MG4 (OOO SKB Stroypribor, Chelyabinsk, Russia) (Figure 2b); viscometer ZM-1001 (Priborelectro LLC, Moscow, Russia); plastometer K-2ZhV (OOO NPP Tochpribor, Rostov-on-Don, Russia) [39,40,41,42,43].

### 2.2. Devices and Methods for Researching Aerated Concrete Mix

To determine the parameters of the aerated concrete mixture and due to the lack of necessary methods, there was a need for special instruments and methods for studying gas release, swelling, and other characteristics described in this section of the work. The device PGV-2A (DSTU, Rostov-on-Don, Russia) operates in automatic control mode. The device parameters are registered by a multipoint automatic potentiometer of the KSP-4 type (OOO RKPO, Moscow, Russia). The volume of the reaction vessel is 2–3 L, the measured volume of the evolved gas is up to 3 L.

**Figure 3:**
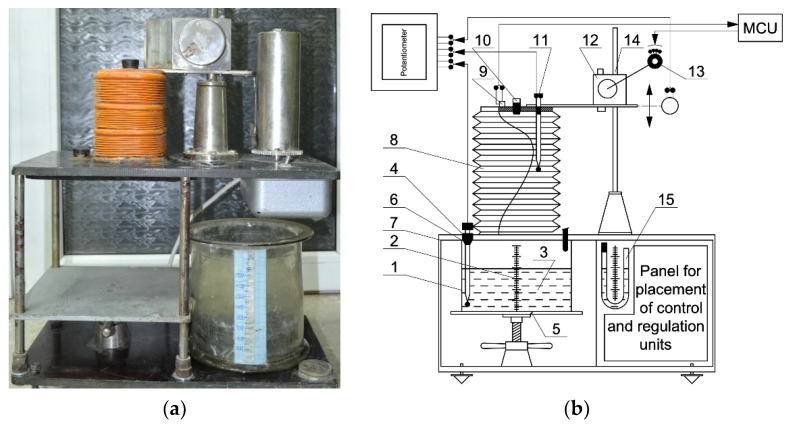

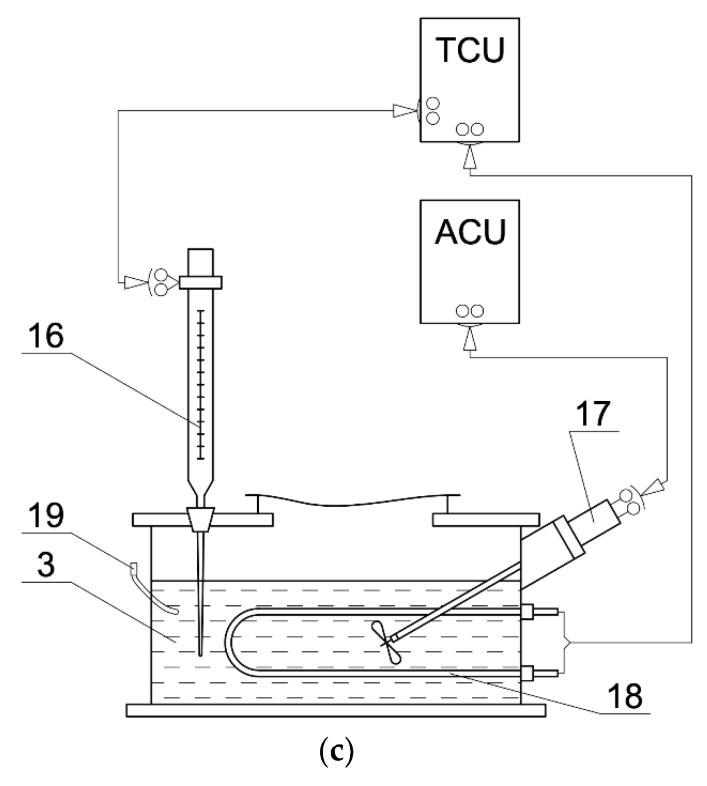
Device for recording the parameters of gas release and swelling of aerated concrete mixtures PGV-2A: (**a**) photo; (**b**) schematic diagram of PGV-2A; (**c**) connection diagram of the heating element and the stirrer of the reaction container.

The device PGV-2A (Figure 3a) consists of a reaction vessel (1) with a scale (2) designed to determine the level of filling and expansion of a substance (3). In addition, there is a thermocouple (4) for measuring the temperature of a substance. A lifting table (5) presses the reaction vessel against the upper base (6). To seal the internal volume, the device is equipped with a rubber gasket (7). A variable measuring container (8) is attached to the upper base of the device, made in the form of a thin corrugated pipe (for example, made of polyethylene). The upper part of the displaced measuring container (8) is closed with a lid, on which a contact pressure sensor (9), a valve (10), and a thermocouple (11) are placed to measure the temperature of the vapor–gas medium in the device above the surface of the substance. To measure the volume of the variable measuring capacity (8), a displacement device (12), a contact pressure sensor (9), a displacement control unit (ACU), and a reversible motor (13) are used, which make it possible to maintain the required pressure in the device when the substance releases gas and changes the temperature of the vapor–gas medium. The volume of the variable capacity (8) is measured by a sensor (14). The control of the internal pressure in the device is carried out by the U-shaped pressure gauge (15) (Figure 3b). To study the kinetics of gas evolution of dispersed gas-forming agents in liquid media at different temperatures, the device is equipped with a special reaction vessel (Figure 3c) containing a contact thermometer (16), a stirrer (17), an electric heating element (18), and a device for introducing a gas-forming agent suspension (19).

On the front control panel of the PGV-2A device, there are controls for internal pressure control, stirring rate control (ACU), and temperature control (TCU) of the reaction medium, as well as the control of the movement of the variable measuring capacitance (MCU).

Using the PGV-2A device, the parameters of the swelling kinetics are determined as follows. First, the test substance is placed in a reaction container installed in the device. Then a thermocouple is introduced into the substance, and the device is sealed. After that, for time points 1, 2, 3…*i*…*p* with a time interval of no more than 15 s, the process, and device parameters are recorded: the temperature of the substance *t*_*B*(*i*)_, the temperature of the vapor–gas medium above the substance *t*_2(*i*)_, the volume of the variable measuring capacitance *V*_*p*(*i*)_, the volume of concrete mix in the container *V*_*B*(*i*)_.

After the completion of the process, the calculation of the parameters of the swelling kinetics of the substance—the swelling coefficient *K*_*B*(*i*)_, the gas diffusion coefficient *D*(*i*), the gas evolution of $V_{\left(i\right)}^{H_{2}}$, the temperature *t*_*B*(*i*)_, for time points (*i*) is carried out according to the following formulas:

(1) $$K_{B\left(i\right)}=\frac{V_{\left(i\right)}^{H_{2}}}{V_{\left(1\right)}^{H_{2}}}$$

(2) $$V_{B\left(i\right)}^{H_{2}}=\frac{293.15\;P_{0}\;\beta_{B\left(i\right)}\left(V_{B\left(i\right)}-V_{B\left(1\right)}\right)}{11,325\;\left(273.15+t_{B\left(i\right)}\right)},\;{\mathrm{cm}}^{3}\;$$

(3) $$V_{D\left(i\right)}^{H_{2}}=293.15\;P_{0}\;\beta_{2\left(i\right)}\;\frac{V_{0\left(i\right)}-V_{B\left(i\right)}}{273.15+t_{2\left(i\right)}}-\frac{V_{0\left(1\right)}-V_{B\left(i\right)}}{273.15+t_{2\left(i\right)}},\;{\mathrm{cm}}^{3}\;$$

(4) $$V_{i}^{H_{2}}=\;V_{B\left(i\right)}^{H_{2}}+V_{D\left(i\right)}^{H_{2}},\;{\mathrm{cm}}^{3}\;$$

(5) $$D_{i}=\frac{V_{D\left(i\right)}^{H_{2}}}{V_{i}^{H_{2}}}$$

where, $V_{0\left(i\right)}$ is the total internal volume of the device (the volume of the reaction and variable measuring capacity), cm^3^; $V_{B\left(i\right)}^{H_{2}}$ is the volume of hydrogen in the cellular concrete mixture, cm^3^; $V_{D\left(i\right)}^{H_{2}}$ is the volume of hydrogen diffused from the cellular concrete mixture, cm^3^; $V_{i}^{H_{2}}$ is the total volume of hydrogen formed during swelling, cm^3^; $P_{0}$ is atmospheric pressure, Pa; $\beta_{2\left(i\right)}=1\;-\;\alpha_{0}\;g_{t}\;\left(273\;+\;t_{2\left(i\right)}\right)$ is the relative content of gases in the vapor–gas medium above the mixture; $\beta_{B\left(i\right)}$ the same, in a mixture; $\alpha_{0}$ = 4.555 cm^3^/(g × deg); $g_{t}$ is the absolute humidity of the gaseous medium (at 100% relative humidity), g/cm^3^ [33,34,35].

## 3. Modeling the Kinetics of Gas Release during the Manufacture of Aerated Concrete

The study considers the fundamental aspects that occur at the level of interaction of various media during gas release and the formation of the aerated concrete structure. The mathematical models of all these processes have been developed. It is proposed to consider aerated concrete mixtures as a system of finite elements, for which one gas bubble is taken. Furthermore, using the apparatus of mathematical physics, the equations of molecular diffusion, and modeling the process of gas release in the emerging aerated concrete, two main hypotheses can be tested:

- Revealing the fundamental-applied connection between the processes of gas release and structure formation of aerated concrete with the performance characteristics of the hardened composite;
- Verification of technological and operational efficiency when using partially hydrated cement in the production of non-autoclaved cellular concrete with improved structural and operational characteristics.

Several processes should first be modeled from the point of view of mathematical physics and then wholly or partially reproduced in the laboratory in a natural experiment. To determine the parameters of the kinetics of gas evolution, it is necessary to stabilize the conditions of the experiment. For this purpose, milk of lime with a density of 1.05–1.2 g/cm^3^ is used as the reaction medium, the temperature of the reaction medium and the stirring rate are maintained constant at a given level [33,34,35].

To determine the parameters of the kinetics of gas evolution, lime milk is poured into the reaction vessel of the PGV-2A device, which is heated to a predetermined temperature. The device is sealed, and then the required amount of the gas-forming suspension is added to the reaction medium, the temperature of the vapor–gas medium *t*_2_(*i*) is recorded, and the internal volume of the device *V_p_* is changed. After the end of the gas evolution reaction, the values of the volume of released hydrogen ($V_{\left(i\right)}^{H_{2}}$) are calculated, reduced to the conditions of *t* = 20 °C, $P_{atm}$= 101,325 Pa, according to the formula:

(6) $$V_{\left(i\right)}^{H_{2}}=\;\frac{293.15\;P_{0}\;\beta_{2\left(i\right)}}{101,325}\left(\frac{V_{i}-V_{p}}{273.15+t_{2\left(i\right)}}-\;\frac{V_{i}-V_{p}}{273.15-t_{2\left(i\right)}}\right)$$

The gas evolution kinetics curve usually has a sigmoid form and can be approximated by a function of the form

(7) $$\frac{V^{H_{2}}}{V_{\max}^{H_{2}}}\;=\;1-\;\exp\left\{-\left[m{\left(\tau -\tau_{ind}\right)}^{n}\right]\right\}$$

where, $V^{H_{2}}$ is an experimental volume of hydrogen formed in the process of swelling; $V_{\max}^{H_{2}}$ is the maximum calculated volume of hydrogen generated during the swelling process; $\tau_{ind}$ is the time of the induction period; *m*, *n*—indicators that determine the curvature of the curve of the kinetics of gas evolution.

The content of actual aluminum in the blowing agent is determined by the formula

(8) $$\alpha =\frac{V_{\max}^{H_{2}}}{1330\;P_{Al}\;}$$

where $P_{Al}$ is the mass of aluminum in the blower, g; 1330 is the volume of hydrogen, cm^3^/g, released during the complete reaction of active aluminum and at *t* = 20 °C and $P_{atm}$= 101,325 Pa.

### 3.1. General Characteristics of the Process of Swelling of Aerated Concrete Mix

The swelling of a cellular concrete mixture is a complex hydrodynamic and physicochemical process that develops over time. It consists of the nucleation and growth of cells due to the reaction of gas evolution, deformation of the mixture at a variable rate under the action of the pressure arising in the cell, vaporization, and condensation of water vapor. At the same time, the temperature, rheological, and diffusion characteristics of the interpore wall and, in general, the entire mixture change. The swelling process is complicated by the boundaries of the expansion in the form and some other conditions.

The process of swelling of aerated concrete mixtures can be conditionally divided into the following periods of the state of aerated concrete mixture—induction, swelling, precipitation, and stabilization of the structure. Each of these periods is characterized by the predominance of individual phenomena that occur during swelling.

The induction period is characterized by slow swelling of the array and an increase in molecular diffusion of hydrogen through the surface of the array to a certain local maximum, the onset of which conditionally indicates the end of this period. The phenomena taking place during this period can be explained as follows. The heterogeneous reaction of the interaction of the liquid (alkaline) phase with aluminum particles having an oxide layer and covered with a stearin film has an induction period that depends on the rate of development of the reactive surface of aluminum particles. The hydrogen released as a result of the reaction diffuses from the surface of a part of aluminum into the liquid phase, saturating it. Due to the high concentration of hydrogen, gas bubbles form at the surface of the particles. At the same time, hydrogen migrates into the depth of the liquid phase and into entrained air bubbles, which are always present in real mixtures. Hydrogen migration also proceeds to the surface of the mixture and into the surrounding atmosphere. A significant hydrogen concentration gradient can explain the predominance of molecular diffusion through the mixture surface on the mixture surface. Supersaturation of the liquid phase with hydrogen is the result of a significant partial pressure of hydrogen in bubbles of a small radius, as well as an insufficient surface of the existing bubbles for the diffusion of molecular hydrogen into them. Achieving the maximum rate of molecular diffusion of hydrogen from the mixture is a conditional transitional moment between the induction period and the intense swelling of the mixture.

The swelling period is characterized by an intensive increase in the volume of the aerated concrete mixture up to a certain maximum value. The phenomena that take place during this period can be explained by the high rate of the gas formation reaction, the increase in the radius of gas bubbles, and their surface area. The limiting factor for swelling is the interpore wall’s structural and rheological kinetic characteristics. A decrease in molecular diffusion is a consequence of a decrease in supersaturation of the liquid phase with hydrogen and saturation of the mixture with gas bubbles. During the period of maximum intensity of swelling, sound emission of the mixture is observed, which indicates the beginning of the formation of defects in the interpore walls in the form of cracks and holes. This moment corresponds to the minimum diffusion of gas through the mixture. The subsequent intensive removal of gas from the mixture is a consequence of the convective diffusion of vapor and hydrogen through defects. The damping of swelling can be explained by convective diffusion of the gas, which reduces the pressure in the bubbles of the mixture. When the gas pressure in the bubbles is balanced and the pressure required to deform the interpore wall, the mixture stops swelling.

The period of precipitation is characterized by a decrease in the volume of the mixture and intense convective diffusion of the vapor–gas mixture from it (gas breakthrough). These phenomena proceed at a variable speed. The sedimentation of the mixture occurs due to the deformation of interpore partitions under the forces of the hydrostatic pressure of the overlying layers. The settling decreases as the internal gas pressure in the bubbles, the resistance stress of the interpore walls to compression, and the hydrostatic pressure of the overlying layers of the mixture are balanced.

The stabilization period is characterized by the absence of a decrease in the volume of the mixture and the high diffusion of gas from it. The phenomena of this period are similar to the phenomena of the previous one. Full stabilization of the mixture is achieved under the condition that the stress of resistance of the interpore walls to collapse exceeds the pressure of the overlying layers. The period of precipitation may be absent if the condition of stabilization of the mixture is met immediately after the period of swelling. The swelling process can be considered completed after the end of gas diffusion through the surface of the mixture.

The considered phenomenological concepts of swelling are the basis of the model of this process [33,34,35].

### 3.2. Physical and Chemical Bases of Phenomena Occurring during Swelling of Aerated Concrete Mixture

Further, based on [33,34,35], the physicochemical foundations of the phenomena occurring during the swelling of an aerated concrete mixture were considered.

#### 3.2.1. Main Characteristics of Cells (Bubbles)

The leading indicators characterizing the dispersed phase are the gas content, disperse composition, and chemical composition of the gas phase. Gas bubbles in cellular concrete are not monodisperse. The average size of the bubbles and their polydispersity largely depend on the specific conditions of production, the rheological and surface properties of the interpore substance, and many other factors. Cells of different sizes in aerated concrete have different values of excess surface energy and therefore have different solubilities or vapor pressures. As a result, in polydisperse systems, particles are redistributed in size in the direction of their enlargement, and the thermodynamic limit of this redistribution is the division of the disperse system into two layers. During swelling, cells in aerated concrete can be enlarged in two ways: coalescence and molecular transfer of gas from small cells to large ones. The simultaneous action of these two mechanisms is also possible.

The stability of gas emulsions with respect to coalescence processes is determined by the interaction energy of gas bubbles and the strength of the two-sided film upon their contact. Therefore, it depends on several conditions: the properties of the dispersion medium and the dispersed phase, the nature of the emulsifier used, the presence of impurities, temperature. The phenomenon of coalescence in the aerated concrete mixture, apparently, prevails at the initial stage of swelling, when the size of the bubbles and the rheological properties of the interpore wall (due to the absence of structural bonds between the particles of the substance) are still small.

In gas-filled disperse systems containing bubbles of various sizes, the size of the bubbles is redistributed due to diffusion mass transfer. The mechanism of this phenomenon is based on the fact that the vapor pressure of small bubbles is greater than that of large ones. The thermodynamic regularities of formation, growth, and distribution of bubbles show that bubbles are more stable, the larger they are. The gas in small bubbles dissolves since the gas concentration in the liquid phase of the material of the interpore wall is lower than the equilibrium one. In contrast, with respect to the largest bubbles, the same liquid phase of the interpore substance is supersaturated, and the gas dissolved in the liquid phase is released into large bubbles. In this case, the largest bubbles grow due to the dissolution of small ones and partially due to the liquid phase of the interpore substance. As a result, the gas concentration in the liquid phase decreases, and the critical size of the bubbles, which are in equilibrium at a given gas saturation of the liquid phase, continuously increases. If gas formation occurs during a chemical reaction in the interpore substance, then the degree of gas supersaturation in the liquid phase reaches large values, significantly exceeding the equilibrium concentrations for small and large bubbles. Therefore, it can be assumed that bubbles of different sizes grow at different rates in an intumescent aerated concrete mixture, and growth is more intense for larger ones.

The gas porosity is:

(9) $$\Pi_{g}={\left(1+\frac{\delta_{av}}{R}\right)}^{-3}$$

where $\delta_{av}$ is the average thickness of the interpore wall per cell; *R* is the average cell radius.

The dependence of the gas porosity of aerated concrete on the average pore radius *R* and the minimum thickness of the interpore partition $\delta_{\min}$ per one pore, and the coordination number *N_k_*, can be represented by the following formula:

(10) $$\Pi_{g}={\left(1+\frac{\delta_{\min}}{R}\right)}^{-3}\;\;\;\frac{\left(N_{k}-2\right)}{N_{k}\left(N_{k}-1\right)}$$

Based on Equations (11) and (12), we obtain the dependence of the minimum thickness of the interpore wall $\delta_{\min}$ on the average thickness of the interpore wall $\delta_{av}$, the average radius *R* of the cell, and the coordination number *N_k_*

(11) $$\delta_{\min}=\;R\left[{\left(\frac{{\left(N_{k}-2\right)}^{2}}{N_{k}\left(N_{k}-1\right)}\right)}^{\frac{1}{3}}\;{\left(1-\frac{\delta_{av}}{R}\right)}^{\frac{1}{3}}-1\right]$$

If the batch volume *V* and the number of cells in the aerated concrete mix *N* are known, then with an average cell radius *R*, the average value of the thickness of the interpore wall $\delta_{av}$ per cell

(12) $$\delta_{av}=\sqrt[{3}]{R^{3}+\frac{3V_{0}}{4\pi N}-R};\;\mathrm{or}\;\frac{\delta_{av}}{R}=\sqrt[{3}]{1+\frac{3V_{0}}{4\pi NR^{3}}-1}$$

at R→0

(13) $$\delta_{av}=\sqrt[{3}]{\frac{3V_{0}}{4\pi N}}$$

The minimum thickness of the interpore partition during swelling of the aerated concrete mixture has a certain critical value, upon reaching which, it is possible to form holes in the interpore partition. This critical thickness depends on the dispersion of the interpore substance and is equal to 3–5 diameters of the coarse component. If we characterize the dispersion of the interpore substance by the specific surface, then the average particle diameter *D_p_* of the interpore substance is equal to

(14) $$D_{p}=\frac{6}{\gamma \;S_{sp}}$$

where γ is the interpore particle density.

However, in natural materials, the thickness of the partitions is much greater than the critical minimum thickness of the interpore partition $\delta_{\min}^{cr}=3D_{p}$, since the packing density of cement and sand particles is insufficient due to their angularity, roughness, and the presence of mixing water in the composition of the mixture. In this regard, it can be assumed that, under the condition $\delta_{\min}$, $\delta_{\min}^{cr}$, the number of defects in interpore walls is equal to

(15) $$N=N_{0}\;\exp\left[-\left(\frac{\delta_{\min}}{\delta_{\min}^{cr}}-1\right)\right]$$

where *N*_0_ is the total number of cells in the mixture (layer).

Considering that as a result of cement hydration (until the stabilization of rheological properties $\tau_{cl}$), a finely dispersed phase of cement neoplasms appears in the composition of the aerated concrete mixture, which affects the deformative properties of the interpore partition, it can be assumed that this circumstance contributes to a decrease in the critical minimum thickness of the interpore partition:

(16) $$\delta_{\min}=\delta_{\min}^{cr1}\;\exp\left(-3\frac{\tau }{\tau_{cl}}\right)\;+\;\delta_{\min}^{cr2}$$

where $\delta_{\min}^{cr1}$ is the initial critical minimum thickness of the interpore wall; $\delta_{\min}^{cr2}$ is the final critical minimum thickness of the interpore wall.

#### 3.2.2. Interaction of a Single Bubble and Interpore Medium

The growth of vapor–gas bubbles in the aerated concrete mixture occurs due to their expansion under the action of the gas released into the bubbles dissolved in the liquid phase or filling them with steam due to mass transfer and heat exchange processes of the bubble and the liquid phase surrounding it.

Both the processes of bubble expansion and filling it with gas or vapor are determined by the level of pressure or resolution in the liquid phase.

The liquid film surrounding the gas bubbles, due to the gas dissolved in the liquid phase, is supersaturated with gas to a concentration determined by the level of gas pressure in the bubble and the solubility coefficient [44], i.e., on the surface of the bubble

(17) $$C_{0}=k_{H}\left(P_{r}T_{r}\right)\;P_{r}$$

where *C*_0_ is the equilibrium concentration; $k_{H}\left(P_{r}T_{r}\right)$ is the Henry solubility coefficient, which depends on temperature and, in general, on the pressure level; *P_r_* is the partial pressure of the gas in the bubble.

The solubility of gases is greatly influenced by temperature: usually, increasing it lowers the solubility. It is known that the solubility of gases in liquids obeys the Clausius–Clapeyron equation. The solubility of gases in aqueous solutions of electrolytes decreases compared to pure water.

If the liquid is saturated to a dissolved gas concentration equal to $C_{\infty }$, and the concentration $C_{0}$ on the bubble surface is lower than $C_{\infty }$, then due to the difference in concentrations $C_{\infty }$–$C_{0}$, a mass flow of the gas dissolved in the liquid into the bubble arises.

At a negative difference $C_{\infty }$–$C_{0}$, the gas in the bubble is dissolved in the liquid phase, and the bubble is compressed.

In the general case, the rate of gas evolution is determined by the rate of gas diffusion in the liquid phase, that is, the rate of transfer to the bubble boundary, the rate of direct release (desorption) of the volume of the gas bubble.

The difference in gas concentrations in the liquid phase leads to the molecular transfer of matter. According to Fick’s first law, the diffusion process is described by the dependence [44]:

(18) $$\frac{dm}{S\;dt}=\;D\left(T,\;p\right)\;{\left(\frac{\partial C}{\partial r}\right)}_{r=R}$$

where, $\frac{dm}{S\;dt}$ is the limiting gas mass flow through a surface unit; *D*(*T*, *p*) is the molecular diffusion coefficient; ${\left(\frac{\partial C}{\partial r}\right)}_{r=R}$ is the derivative of the concentration along the normal to the bubble surface.

The rate of substance transfer at a given temperature and pressure is proportional only to the concentration gradient near the interface.

The value of the concentration derivative in Equation (21) at a fixed interface is determined from the solution of the molecular diffusion equation

(19) $$\frac{\partial C}{\partial r}=D\left(\frac{\partial C}{\partial r}+\frac{2}{r}\;\frac{\partial C}{\partial r}\right)$$

at *τ* = 0 *r* = *R*, at *τ* = ∞ *r* → ∞, *C* = *C*_∞_ at *τ* > 0 *r* = *R*, *C* = *C*_0,_ at *τ* = ∞, *r* → ∞, *C* = *C*_∞_ (20)

The solution of Equation (19) under the initial and boundary Conditions (20) makes it possible to determine the concentration gradient at the bubble boundary

(21) $${\left(\frac{\partial C}{\partial r}\right)}_{r=R}=\;\left(C_{\infty }-C_{0}\right)\;\left(\frac{1}{R}+\frac{1}{\sqrt{\pi D\tau }}\right)$$

Dependences (18) and (21) the mass flow of gas through a single area, which is determined by the process of molecular diffusion in a liquid, can be found

(22) $$\frac{\partial m}{\partial \tau }=DS\left(C_{\infty }-C_{0}\right)\left(\frac{1}{R}+\frac{1}{\sqrt{\pi D\tau }}\right)$$

With a time-varying gas concentration in liquid *C*_∞_(*τ*) and *C*_0_(*τ*), Equation (25) takes the form

(23) $$\frac{\partial m}{\partial \tau }=DS\left(T\right)\;\left[C_{\infty }\left(\tau \right)-C_{0}\left(\tau \right)\right]\;\left(\frac{1}{R}+\frac{1}{\sqrt{\pi D\tau }}\right)$$

In the case of a vapor–gas bubble, the rate of its growth or dissolution depends, in addition to diffusion, on heat transfer, which is described by the control of heat transfer during liquid evaporation or vapor condensation. The equilibrium vapor pressure (vapor pressure) over a concave liquid surface (with radius R) is lower than for a flat interface. It can be determined from the Kelvin–Helmholtz relation, which for the case $\rho_{l}$, $\rho_{v}$ has the form

(24) $$P_{v}=P_{v}^{1}\;\exp\left(-\frac{2\sigma }{R}\;\frac{1}{\rho_{l}BT}\right)$$

where $P_{n}^{1}$ is the vapor pressure over a flat surface.

For capillaries with a radius greater than 10^−5^ cm, the saturation vapor pressure above the meniscus in the capillary is equal to the saturation vapor pressure over a flat surface with an accuracy of 1%. For the entire set of bubbles in the aerated concrete mixture, the amount of gas released into the bubble is equal to

(25) $$\frac{\partial m}{\partial \tau }=\;D4\pi NR^{2}\left[C_{\infty }\left(\tau \right)-C_{0}\left(\tau \right)\frac{1}{R}-\frac{1}{\sqrt{\pi D\tau }}\right]$$

#### 3.2.3. Conditions for the Deformation of the Gas Mixture

Swelling of the mass of aerated concrete mixture occurs as a result of deformation of the interpore wall under the action of gas pressure in the cells, and the sediment is the result of deformation of the interpore material under the action of hydrostatic pressure from the overlying layers of the aerated concrete mixture.

According to the theory of the energy of shape change of a plastic body, the specific energy of shape change for any point remains constant in the plastic state of matter. Based on this, when deforming an array of aerated concrete, you can write the following expression for the pressure of the vapor–gas medium in the cells:

(26) $$P_{0}=P_{\sigma }+P_{h}+P_{mn}+P_{v}$$

where *P*_0_ is atmospheric pressure; $P_{\sigma }$—pressure from the action of surface energy forces at the boundary of two phases; *P_h_*—hydrostatic pressure from the overlying layers of aerated concrete mixture; *P_mn_* is the pressure required for plastic deformation of the interpore wall; *P_v_* is the pressure caused by the limited swelling of the mixture in the mold and the constraint of cell movement.

The condition of swelling and settling of an aerated concrete mixture and any elastic-plastic system can be justified by the position of the problem of motion stability. The effect of the loss of stability of the interpore partition with an increase in the internal pressure in the cell is the appearance of swelling deformations that occur when a certain critical value of the internal pressure in the cell is exceeded, that is:

(27) $$P_{c}-P_{0}-P_{\sigma }-P_{h}\geq P_{mn}^{B}$$

When the mixture settles, there is a loss of stability of the interpore wall, which occurs under the action of the hydrostatic pressure of the overlying layers when the condition

(28) $$P_{c}-P_{0}-P_{\sigma }-P_{h}<P_{mn}^{0}$$

Cellular concrete mixtures and their interpore partitions under applied stresses not exceeding the limiting shear stress *τ*_0_ behave like a solid body, and when these stresses are exceeded, they irreversibly flow with a strain rate *dv*/*dy* due to the plastic viscosity of the mixture and can be described by the Shvedov–Bingham

(29) $$\tau =\tau_{0}+\eta \frac{dv}{dy}$$

In an utterly plastic state of a polydisperse medium with spherical inclusions, the pressure causing such a state is expressed by the dependence

(30) $$P_{mn}=\pm 4\tau \;\ln\frac{r+\delta }{r}$$

The formula determines the critical value of swelling pressure:

(31) $$P_{mn}^{S}=4\tau_{0}\;\ln\frac{r+\delta }{r}$$

and the critical value of sediment pressure:

(32) $$P_{mn}^{0}=-4\tau_{0}\;\ln\frac{r+\delta }{r}$$

The pressure in the bubble from the action of surface energy forces at the bubble–interstitial wall boundary is determined by the formula:

(33) $$P_{\sigma }=\frac{2\sigma }{R}$$

where σ is the surface tension of the liquid at the phase boundary.

The hydrostatic pressure from the overlying layers is:

(34) $$P_{h}=\gamma_{mn}\;g\left[1-{\left(1+\frac{\delta }{R}\right)}^{-3}\right]\;\left(H-h\right)$$

where *γ_mn_* is the average density of the interpore wall; σ is the surface tension of the liquid at the phase boundary; *H* is the height of the aerated concrete mixture; *h* is the height from the bottom of the mold to the layer under study; *g* = 9.81 m/s^2^.

### 3.3. Modeling the Kinetics of Swelling of Aerated Concrete Mix

**Figure 4:**
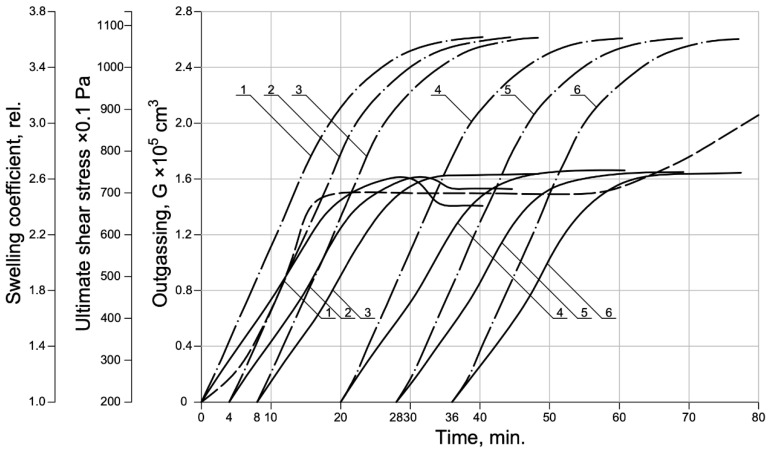
Kinetics of swelling of an aerated concrete mixture (simulation): 1–6 are experiment numbers.

The presented mathematical model [33,34,35,45,46,47,48] was used to simulate the process of swelling of an aerated concrete mixture under conditions that correspond to the hypotheses of this study, to confirm the assumptions made about increasing the homogeneity of material properties, due to the swelling process during the start of coagulation of the colloidal fraction of the liquid phase of the mixture (Figure 4).

For this purpose, the conditions of the process of swelling of the aerated concrete mixture were modeled, under which the time-varying parameters were the outgassing of the gasifier *G*(j), the limiting shear stress of the interpore substance *τ*_0_(j), and the temperature of the mixture *T*_0_ was constant and equal to 40 °C.

The initial data for the calculation according to the model were also:

1. Mixing volume $V_{0}$ = 10^5^ cm^3^.
2. Sectional area of the array *S*_0_ = 2.5 × 10^3^ cm^2^.
3. Volume of gas saturation of the mixture (nuclei) φ = 0.27%.
4. Atmospheric pressure *P*_0_ = 1,013,250 dynes/cm^2^.
5. Molecular diffusion coefficient D = 4.8 × 10^−5^ cm^2^/s.
6. Henry coefficient k_H_ = 1.1618 × 10^−10^ (cm^3^/cm^3^) × (cm^2^/dyne).
7. Partial pressure of water vapor *P*_n_ = 76,196.4 dynes/cm^2^.
8. Surface tension of water σ = 70 erg/cm^2^.
9. Average density of interpore substance γ_mn_ = 1.762 g/cm^2^.
10. W/T—mixture ratio = 0.5.
11. Density of water $\rho_{l}$ = 1 g/cm^3^.
12. The average density of the solid phase 𝜌_T_ = 2.874 g/cm^3^.
13. Coordination number of cells *N*_k_ = 12.
14. Free fall acceleration g = 980 cm/s^2^.
15. The thickness of the diffuse film δ = 10^−4^ cm.
16. Number of layers i = 10.
17. The number of analyzed time intervals j = 40.
18. Time interval Δ*τ* = 60 s.
19. The initial concentration of hydrogen in the liquid phase is *C_∞_*(0) = 0.
20. The average initial radius of steam–air bubbles (nuclei) of the mixture is R(i,0) = 10^−2^.
21. Coefficient M = 10^−9^ s;
22. The minimum critical thickness of the interpore wall at the beginning of the process per cell $\delta_{\min}^{kp1}$ = 5 × 10^−3^ cm.
23. The minimum critical thickness of the interpore wall 20 min after mixing the mixture with water $\delta_{\min}^{kp2}$ = 5 × 10^−3^ cm.

Swelling modeling was carried out for six experiments, differing in the moment of the beginning of the gas evolution of the blowing agent in the mixture.

An analysis of the simulation presented in Figure 4 shows that, under these experimental conditions, a shift in the onset of gas evolution by 4 and 8 min (experiments 1, 2, and 3), relative to the moment of combining the mixture with water, leads to a change in the swelling coefficient of the mixture by more than 8%. Furthermore, an increase in the start time of gas evolution (experiments 4, 5, and 6) leads to stabilizing the swelling parameters. Furthermore, in all experiments in the process of swelling, the heterogeneity of the parameters of individual layers of the aerated concrete mixture is observed, which is determined by the magnitude of the hydrostatic pressure from the overlying layers of the mixture, and the degree of convective diffusion of hydrogen, leading to sedimentation and compaction of the concrete.

The observed inhomogeneity of the properties of the expanded aerated concrete mixture depends on the correspondence between the kinetics of microstructure formation of the interpore substance and the gas release of the blowing agent, and decreases with a shift at the beginning of the kinetics of the blowing out of the blowing agent, in the region of stabilization of the rheological properties of the interpore wall.

The simulation of the swelling of aerated concrete mixture made it possible to identify two practical directions for implementing the provisions of the hypothesis: the first is the use of a blowing agent with a long induction period, the second is the use of pre-hydrated cement as a binder.

## 4. Results and Discussion

### 4.1. Study of the Influence of Technological Factors on the Structure Formation of Aerated Concrete, Analysis of the Kinetics of the Properties of the Interpore Substance of the Aerated Concrete Mix

The interpore wall of the aerated concrete mix consists of a multi-component substance. The change in the rheological characteristics is determined mainly by the kinetics of the structure formation of the binder.

To study the swelling processes of aerated concrete mixtures, of particular interest is the initial period of structure formation of the binder, and the period of intense hydration, coinciding in time with the stage of formation of the cellular structure.

To assess the kinetics of the rheological properties of the material of the interpore wall of the aerated concrete mixture, the change in its ultimate shear stress (*τ*_0_), plastic viscosity (η), and plastic strength (*P_m_*) in time equal to the swelling period (0–80 min) was studied.

**Figure 5:**
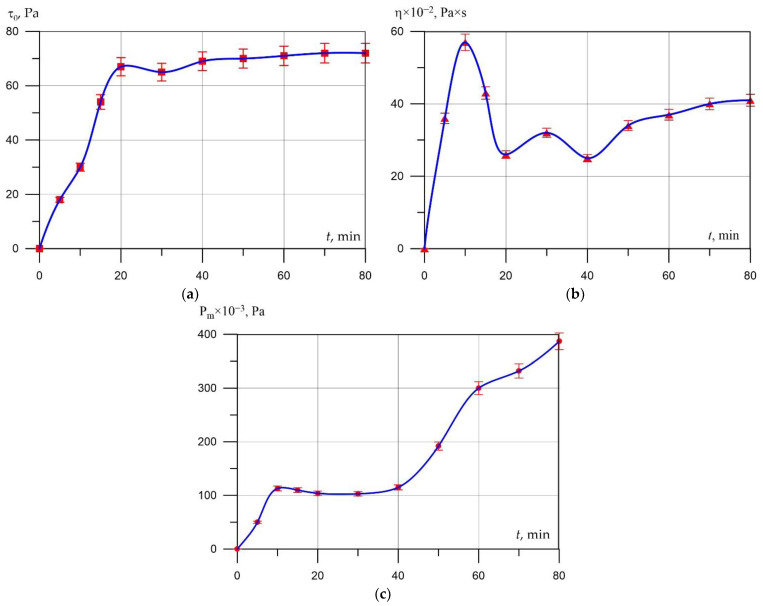
Kinetics of structure formation of the interpore substance of aerated concrete mix (spline approximation): (**a**) ultimate shear stress; (**b**) plastic viscosity; (**c**) plastic strength.

The test procedure was adopted as follows. Raw materials (Portland cement and ground sand *S_sp_* = 3000 cm^2^/g) were mixed with water (W/T = 0.5), for 3 min. The mass (at *t* = 35 °C) was placed in a viscometer and, upon reaching a given point in time, the values *τ*_0_ and η were determined. The experiment was repeated 3 times. The results of the experiment are shown in Figure 5.

An analysis of the obtained curves of the kinetic rheological properties of the mixture shows that in the first minutes after mixing, there is a sharp increase in the values of the ultimate shear stress, observed up to the 20th minute, followed by a slight decline and stabilization. The rate of change of η reaches its maximum value after 10 min, then it decreases by the 20th minute and stabilizes after 70 min (to the value of 40 × 10^−2^ Pa×s) (Figure 5b). The curve of change in the plastic strength of the interpore material has the only similarity—a rapid increase up to 10 min. Then stabilization and after 40 min a rapid increase. This character of the *τ*_0_ and *P_m_* curves is explained by the intensive processes of hydration of clinker minerals in the initial period of structure formation, the appearance of hydrate neoformations such as ettringite and Ca(OH)_2_ on their surface, and the appearance of coagulation bonds between the particles of the hardening system (Figure 5a,c), which does not contradict the data [49,50].

The fact that the values of *τ*_0_ and Pm stabilize in the period of 20–40 min is explained by the saturation of the liquid phase of the Ca(OH)_2_ interpore substance, fine-grained cement neoplasms, and the appearance of a binder film of hydrated neoplasms on the surface of the grains, which prevents the development of structure formation of the cement system. The instability of the parameters of the hydration kinetics of cement in the initial period, and the impossibility of operational control of hydration, leads to a change in the kinetics of swelling of the aerated concrete and the heterogeneity of the final properties. It is possible to reduce the influence of the instability of the initial period of structure formation on the process of swelling of the aerated concrete mixture, if the gas release of the blowing agent begins during the period of stabilization of the properties of the cement system. This can be achieved by introducing pre-hydrated cement into the aerated concrete mixture or by holding the mixture until the blowing agent is introduced.

### 4.2. Study of the Influence of the Time of Preliminary Hydration of the Binder on the Rheological Properties of the Interpore Substance, the Swelling Kinetics, and the Properties of Aerated Concrete

The alignment characterizes the traditional casting technology in a time of three kinetic phenomena—intensive hydration of the binder, changes in the rheological properties of the interpore material, and the gas evolution reaction of the aluminum blowing agent, which have a decisive influence on the swelling kinetics and physical and mechanical properties of aerated concrete. The study on the phenomenological model, carried out in the previous section, revealed that if the outgassing occurs during the period of intensive hydration of the binder (up to 30 min from the start of mixing), then the swelling kinetics is unstable, and a slight change in the outgassing parameters, rheological, and other properties lead to significant changes in the swelling process.

To achieve increased physical and mechanical properties, an optimal combination of the kinetics of hydration phenomena, microstructure formation of the interpore wall, and gas release is necessary. To create favorable conditions for swelling, cement previously mixed with water and aged for a given time, was introduced into the composition of the aerated concrete mixture as its component. At the same time, the process of cement structure formation reached the stage of coagulation of hydrate neoplasms, and the rheological properties stabilized.

Preliminary studies of the influence of the water-cement ratio W/C (0.3–0.6) on the regularities of the kinetics of the rheological properties of the cement paste showed that the time for the start of stabilization of the limiting shear stress with an increase in W/T increases from 17 to 24 min. For the convenience of work in further experiments, W/C = 0.5 was taken.

**Figure 6:**
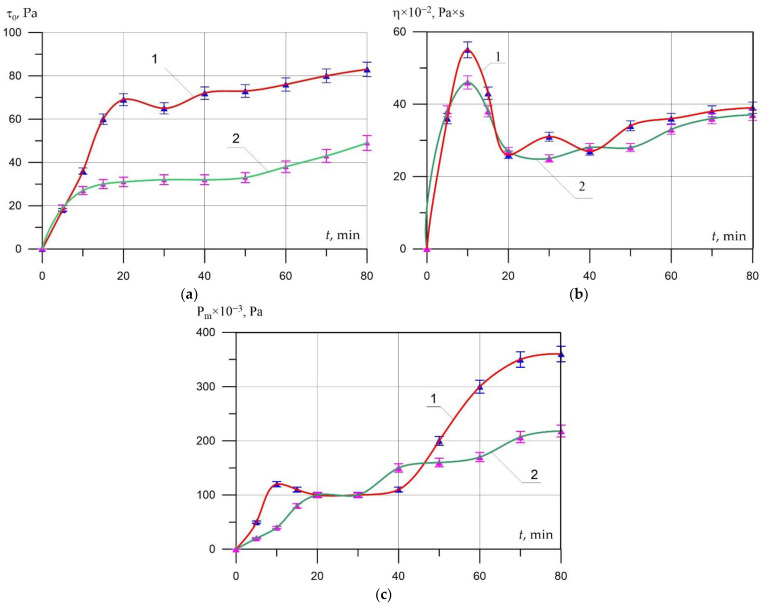
Influence of preliminary hydration of the binder on the rheological characteristics of the interpore material (1—non-porous mixture of the control composition; 2—non-porous mixture on pre-hydrated binder): (**a**) ultimate shear stress; (**b**) plastic viscosity; (**c**) plastic strength.

As a result of tests (Figure 6b), it was found that with an increase in the pre-hydration time of the binder, the value of plastic viscosity stabilizes over time and the value of the initial peak of plastic viscosity observed in the control composition decreases significantly. This is explained by the fact that in the cement system with preliminary hydration of the cement, by the time of testing, phenomena have already occurred that lead to the appearance of a peak on the plastic viscosity curve. That is, it is a reflection of the properties of cement. The decrease in the magnitude of the ultimate shear stress (Figure 6a) in pre-hydrated cement systems is explained by the mechanical destruction of the initial contacts between cement particles covered with shells of hydrated neoplasms. The magnitude of the reduction in the ultimate shear stress during the curing of the cement system is determined by the degree of completion of the formation of the primary shell by the testing time. As for the change in plastic strength (Figure 6c), in the interval from 0 to 40 min, its values for both compositions vary from 20 to 150 Pa. However, starting from 50 min, a sharp increase in plastic strength is observed in the non-porous mixture of the control composition.

**Figure 7:**
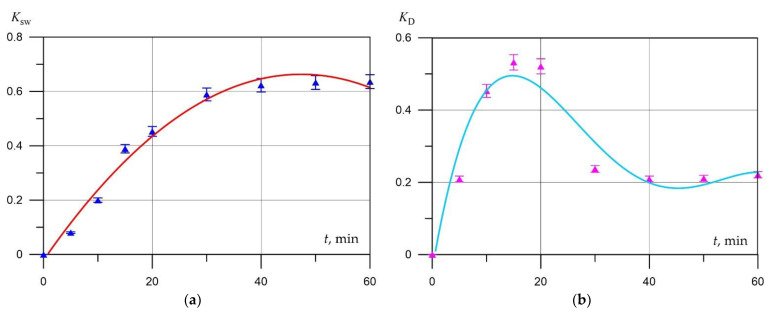
Kinetics of (**a**) swelling of mixture; (**b**) gas diffusion from a mixture, with pre-hydrated cement.

The results of swelling of an aerated concrete mixture (Figure 7a,b) of the same composition, which do not differ in the time of preliminary hydration of the cement, show that with an increase in the holding time to 30 min, an intensive increase in the swelling coefficient is observed, in the range from 30 to 60 min, the value of the swelling coefficient stabilizes. The maximum value of the diffusion coefficient was fixed at 15 min, and starting from the 20th minute, it decreases and stabilizes from the 30th minute, which is the result of an increase in the gas-retaining capacity of the aerated concrete mixture and a change in the rheological properties of the interpore wall material.

To assess the effectiveness of the pre-hydration time of the binder on the mechanical and physical characteristics of aerated concrete, the coefficient of constructive quality (CSQ) [51,52] was calculated:

(35) $$\mathrm{CSQ}=\frac{R_{b}}{\rho_{av}}\;\;,\;\mathrm{MPa}/\mathrm{kg}\times m^{3}$$

where, *R_b_* is compressive strength, MPa; *ρ_av_* is average density, kg × m^3^.

The values of the average density of aerated concrete and its compressive strength (Table 4) show that the maximum reduction in average density by 29% (from 695 to 497 kg/m^3^) and thermal conductivity by 31%, as well as an increase in CSQ, is achieved with a pre-hydration time of 20–30 min. At the same time, according to the CSQ indicator, it is noticeable that the dynamics of strength reduction in this pre-hydration time interval is the smallest CSQ is 7–13% higher compared to other time intervals.

A further increase in the pre-exposure time is impractical since it worsens the quality of the structure. Thus, the determination of the adequate time of preliminary hydration of cement at a given material strength can be determined from the corresponding values of its average density and thermal conductivity.

In order to assess the contribution of the study to the base of world science, it is necessary to determine its place in comparison with the results obtained by other authors. The study has a theoretical and applied character and consists of two main aspects. The first is the filling of research gaps, expressed in the study of the very nature of the process of outgassing and modeling of these processes to obtain more accurate theoretical knowledge and develop existing ideas about the processes occurring during the formation of the microstructure of aerated concrete. The second aspect is the filling of technological deficiencies, expressed in the determination of quantitative and qualitative aspects and values of the recipe and technological parameters that affect the quality of the final material—non-autoclaved aerated concrete of a new level, and obtaining effective building structures, buildings, and structures from them. In comparison with the researchers who previously carried out work to determine the nature of the formation of the aerated concrete structure, in particular with the works [34,35], more accurate data on the process of formation of the aerated concrete structure were established, the nature of the porosity that occurs when using modern building materials and initial components was clarified in comparison with the work carried out about 40 years ago and taking into account all the achievements of modern science. From the point of view of technology, our results have led to an increase in the qualitative and quantitative characteristics of the resulting aerated concrete, expressed in a decrease in average density by 29% and thermal conductivity by 31%, and at the same time in the smallest loss of strength, confirmed by the highest coefficient of structural quality (the difference was up to 13%), compared with the works [34,35].

## 5. Conclusions

Based on theoretical analysis, experimental studies, and the mathematical model of the swelling process, which take into account the convective and molecular diffusion of hydrogen from the mixture and the conditions of swelling, precipitation, and stabilization of the mixture, the increase in the uniformity of the level of swelling of the aerated concrete mixture was developed.

An improved method for the manufacture of aerated concrete is proposed, which comprises preliminarily hydrated for 20–30 min cement into the composition of the aerated concrete mixture. The method provides an improvement in the gas-holding capacity and an increase in the swelling of the mixture, a decrease in the average density of aerated concrete up to 29%, and an improvement in heat-shielding properties up to 31%. At the same time, the smallest dynamics of the decrease in the strength properties of aerated concrete were observed, which is confirmed by a large CSQ of up to 13%.

Aerated concrete has been obtained that meets the requirements of environmental friendliness and economic efficiency, and has improved mechanical and physical characteristics. The effectiveness of the use of production waste in the form of partially hydrated cement in the compositions of such aerated concrete has been proven, thereby reducing the cost of production of aerated concrete and construction in general, amounting to about 15%.

Thus, the environmental effect of our proposals is to reduce the burden on the environment through the use of waste-free technology. Such technology is the addition of partially hydrated cement, i.e., a waste from aerated concrete production, in a new production. At the same time, the environmental effect develops into an economic one, which is expressed in a reduction in the cost of raw materials, which is especially important for manufacturers.

## Figures and Tables

**Table 1:** Physico-mechanical characteristics and chemical composition of Portland cement CEM I 42.5N.

Property	Value
Physical and mechanical	
Compressive strength at the age of 28 days, MPa	44.7
Setting time, min	
- start	155
- end	220
The fineness of grinding, passage through a sieve N 008, %	96.7
Specific surface, m^2^/kg	331
Normal density of cement paste, %	23.5
Chemical	
Loss on ignition, wt%	0.70
Silicon oxide content (SiO_2_), %	20.89
Content of aluminum oxide (Al_2_O_3_), %	4.72
Iron oxide content, (Fe_2_O_3_), %	4.32
Content of calcium oxide (CaO), %	63.27
Mass fraction of magnesium oxide (MgO), %	2.45
Mass fraction of sulfuric anhydride (SO_3_), %	2.81
Mass fraction of alkali oxides in terms of Na_2_O, %	0.60
Content of free calcium oxide (CaO_fr_), %	0
Mass fraction of chloride ion (Cl^−^), %	0.038
Insoluble residue, %	0.20

**Table 2:** Physical characteristics of fine aggregate.

Grain Composition							Pass Through a Sieve Mesh 0.16, wt%	Size Modulus	Content of Dust and Clay Particles, %	True Density, kg/m^3^	Bulk Density, kg/m^3^
Sizes of Sieve Openings, mm											
Private and Total Residues on Sieves, %											
10	5	2.5	1.25	0.63	0.315	0.16					
0	0	0.17	1.39	8.86	45.80	41.03	2.49	1.66	1.1	2650	1438
		0.17	1.56	10.42	56.21	97.25	99.74				

**Table 3:** Physical properties and chemical composition of aluminum powder.

Covering Capacity on Water, cm^2^/g	Residue on Sieve 0.08, %	Chemical Composition, %							Buoyancy, %
		Active Aluminum	Impurities						
			Fe	Si	Cu	Mn	Moisture	Fat	
7000	1.0	-	0.4	0.3	0.05	0.01	0.2	3.5	80

**Table 4:** Physical and mechanical properties of aerated concrete and their change depending on the time of preliminary hydration of the binder.

Num	Pre-Hydration Time, Min	Average Density ρ_av_, kg/m^3^	Decrease in Average Density Δ_ρ__av_, %	Compressive Strength R_b_, MPa	Reduction in Compressive Strength Δ_Rb_, %	Coefficient of Thermal Conductivity λ, W/m·K	Reduction of the Coefficient of Thermal Conductivity Δ_λ_, %	CSQ, MPa/kg × m^3^ × 10^−3^
1C	-	695	0	4.98	0	0.154	0	7.2
2C	10	630	9.4	4.50	9.6	0.145	5.8	7.1
3C	15	564	18.8	4.09	17.9	0.130	15.6	7.3
4C	20	497	28.5	3.84	22.9	0.115	25.3	7.7
5C	30	508	26.9	3.87	22.3	0.106	31.1	7.6
6C	40	548	21.2	3.95	20.7	0.113	26.6	7.2
7C	50	569	18.1	4,01	19.5	0.124	19.5	7.0
8C	60	613	11.8	4.16	16.5	0.151	1.9	6.8

## Data Availability

The study did not report any data.
